# A Review of Impaired Neuroplasticity in Schizophrenia Investigated with Non-invasive Brain Stimulation

**DOI:** 10.3389/fpsyt.2016.00045

**Published:** 2016-03-29

**Authors:** Apoorva Bhandari, Daphne Voineskos, Zafiris J. Daskalakis, Tarek K. Rajji, Daniel M. Blumberger

**Affiliations:** ^1^Centre for Addiction and Mental Health, Temerty Centre for Therapeutic Brain Intervention, Campbell Family Mental Health Research Institute, University of Toronto, Toronto, ON, Canada; ^2^Department of Psychiatry, Centre for Addiction and Mental Health, Temerty Centre for Therapeutic Brain Intervention, Campbell Family Mental Health Research Institute, University of Toronto, Toronto, ON, Canada

**Keywords:** schizophrenia, transcranial magnetic stimulation, motor cortex, neuroplasticity, long-term potentiation

## Abstract

**Background:**

Several lines of evidence implicate dysfunctional neuronal plasticity in the pathophysiology of schizophrenia (SCZ). Aberrant glutamatergic and gamma amino-­butyric acid neurotransmission are thought to underlie core cognitive deficits and negative symptoms of SCZ. Non-invasive brain stimulation (NIBS) allows for the *in vivo* study of cortical plasticity and excitability at the systems level of the human motor cortex. This review will focus on summarizing the available neurophysiological evidence for impaired motor cortical plasticity in SCZ assessed by NIBS.

**Methods:**

A search of MEDLINE, Embase, and PubMed was performed on the use of NIBS techniques to investigate neuroplasticity in the motor cortex of SCZ patients. The relevant articles were summarized.

**Conclusion:**

Our review of the literature reveals evidence for disrupted neuroplasticity in SCZ and its close association to alterations in cortical inhibition and dysfunctional intracortical connectivity. Further investigations are required to elucidate the neurobiological mechanisms that underlie dysfunctional plasticity in SCZ in order to develop more targeted therapeutic interventions for SCZ patients.

## Introduction

Schizophrenia (SCZ) is a serious neuropsychiatric illness characterized by a complex phenotype including positive, negative, and cognitive symptoms. This disorder affects nearly 1% of the population (1) incurring substantial societal, economic, and personal costs. Despite its societal burden the pathophysiological underpinnings of SCZ remain poorly understood. Several lines of evidence implicate disturbed neuroplasticity in the pathophysiology of SCZ incorporating alterations in neurotransmitter systems and cortical connectivity with clinical observations of cognitive deficits and negative symptoms (2–7).

Neuroplasticity refers to the brains ability to reorganize and generate new neuronal pathways in response to internal and external stimuli. Neuroplasticity is contingent on neuronal micro- and macro-connectivity and activity-dependent changes in neuronal synaptic strength (8, 9). The strengthening of neuronal connections in highly activated pathways is termed long-term potentiation (LTP). The weakening of inadequately activated neuronal pathways is termed long-term depression (LTD). LTP and LTD are thought to be the neural mechanisms that underlie learning and memory (10).

Glutamatergic *N*-methyl-d-aspartate (NMDA) receptors play a crucial role in the molecular processes of LTP and LTD (11). Several studies have linked NMDA receptor hypofunction to aberrant LTP and LTD in SCZ. Specifically, the administration of NMDA receptor antagonists, such as ketamine and phencyclidine, provides support for the theory of NMDA receptor hypofunction and impaired plasticity in SCZ (4, 12–14). In recent literature, dysfunction of glutamatergic NMDA receptors with both a hypoglutamatergic and a periodic hyperglutamatergic state in SCZ patients has been discussed (2, 15, 16).

Gamma amino-butyric acid (GABA) also plays a critical role in the modulation of synaptic plasticity (17). The association between glutamate and GABA is well established. NMDA receptors are known to modulate the firing rate of GABAergic interneurons (18). Evidence for disturbed GABAergic neurotransmission in SCZ has been demonstrated through several postmortem studies which have shown both decreased density of GABAergic interneurons in multiple cortical regions as well as alterations in the GABA-synthesizing enzyme glutamic acid decarboxylase (3, 19–24).

Non-invasive brain stimulation (NIBS) allows for the investigation of cortical excitability and plasticity at the systems level of the human motor cortex (7). In combination with neuroimaging techniques, NIBS can be used to explore other cortical regions related to mental disorders. NMDA receptor function and calcium homeostasis have proven to be critical for cortical plasticity induction following NIBS in healthy subjects (25–28). This makes NIBS an ideal investigatory tool to explore NMDA receptor-dependent cortical plasticity in SCZ patients (29).

Herein we will briefly examine five NIBS techniques before summarizing the neurophysiological evidence for motor cortical plasticity deficits in SCZ. Following this, the inherent limitations of the summarized studies are discussed.

## Non-invasive Brain Stimulation

This review will discuss the following five NIBS techniques, distinguished from each other by their unique mode of action: repetitive transcranial magnetic stimulation (rTMS), paired-associative stimulation (PAS), use-dependent plasticity (UDP), transcranial direct current stimulation (tDCS), and theta-burst stimulation (TBS) (refer to Table 1 for an overview).

**Table 1:** **NIBS-induced cortical plasticity investigated in the motor cortex of SCZ patients**.

Stimulation	Plasticity	Patients	Main result	Reference
1-Hz rTMS (left M1)	LTD-like	16 medicated SCZ	Reduced LTD-like plasticity in medicated and unmedicated patients and impaired cortical inhibition	Fitzgerald et al., 2004 (36)
		10 unmedicated SCZ		
		18 HC		
1-Hz rTMS (left PMC)	LTD-like	12 SCZ	Reversed plasticity (LTP-like) plasticity in SCZ patients unlike controls and reduced cortical inhibition	Oxley et al. (42)
		12 HC		
PAS 25 (left M1)	LTP-like	15 SCZ	Deficient LTP-like plasticity in SCZ patients associated with impaired motor learning and reduced cortical inhibition	Frantseva et al. (47)
		15 HC		
UDP	LTP-like	14 medicated SCZ	Both patient groups failed to show an LTP-like plasticity response indicating a disturbed cortical reorganizational process	Daskalakis et al. (9)
		6 unmedicated SCZ		
		20 HC		
Anodal tDCS (left M1)	LTP-like	9 RO-SCZ	ME-SCZ, not RO-SCZ, demonstrated impaired LTP-like plasticity following anodal-tDCS indicating a disease-course-dependent effect on plasticity; reduced cortical inhibition was only enhanced in RO-SCZ after anodal tDCS	Hasan et al. (52)
		13 ME-SCZ		
		22 HC		
Cathodal tDCS (left M1)	LTD-like	21 SCZ	SCZ patients showed reduced LTD-like plasticity and enhanced cortical inhibition	Hasan et al. (53)
		21 HC		
Cathodal tDCS (left M1)	LTD-like	18 SCZ	SCZ patients demonstrated reduced LTD-like plasticity on the stimulated hemisphere and abolished LTD-like plasticity on the non-stimulated hemisphere indicating an association between impaired plasticity and connectivity	Hasan et al. (54)
		18 HC		
Cathodal tDCS (left M1)	LTD-like	15 SCZ	SCZ patients and first-degree relatives demonstrated abolished LTD-like plasticity in the stimulated hemisphere. SCZ patients showed abolished LTD-like plasticity in the non-stimulated hemisphere, whereas first-degree relatives displayed reversed plasticity	Hasan et al. (55)
		12 Relatives		
		20 HC		
Unilateral cathodal tDCS (left M1), Bilateral (cathodal left M1, and anodal right M1)	LTD-like (left M1)	10 SCZ	SCZ patients failed to show LTP and LTD-like plasticity following both unilateral and bilateral stimulation paradigms. HC demonstrated LTD-like plasticity (cathodal left M1) after unilateral stimulation and LTP-like plasticity induction (anodal right M1) following bilateral stimulation.	Hasan et al. (56)
		10 HC		
	LTP-like (right M1)			
TBS (left M1)	LTP-like (cTBS300)	10 SCZ	SCZ patients showed impaired LTD-like plasticity following cTBS600 stimulation and reversed plasticity following cTBS300	Hasan et al. (60)
		10 HC		
	LTD-like (cTBS600)			

*M1, primary motor cortex; SCZ, schizophrenia patients; ME-SCZ, multi-episode schizophrenia patients; RO-SCZ, recent-onset schizophrenia patients; HC, healthy control subjects; tDCS, transcranial direct current stimulation; Relatives, unaffected first-degree relatives of schizophrenia patients; PAS, paired-associative stimulation; rTMS, repetitive transcranial magnetic stimulation; PMC, primary motor cortex; UDP, use-dependent plasticity; TBS, theta burst stimulation; LTP, long-term potentiation; LTD, long-term depression*.

While all reviewed NIBS techniques have been shown to induce LTP-like and LTD-like plasticity there are key differences in their mechanisms of response. PAS induces a form of heterotopic plasticity as it is dependent on the synchronous activation of two different inputs converging on the same cell (30–32). In contrast, rTMS, tDCS, and TBS induce a form of homeotopic plasticity caused by the repeated activation of the same input (i.e. set of synaptic connections) and the effects are dependent on the frequency of stimulation (30, 33).

### Repetitive Transcranial Magnetic Stimulation

Transcranial magnetic stimulation (TMS) is a non-invasive tool that allows for the study of cortical excitability and plasticity *in vivo*. TMS relies on the principle of electromagnetic induction. A TMS coil is used to produce a focal magnetic field that in turn induces electrical current in the cortical tissue, resulting in transsynaptic activation of cortical neurons (7). The cortical plasticity induced by NIBS is demonstrated by alterations in motor cortical excitability (17, 34). With regards to the studies described below, changes in cortical excitability following NIBS have been assessed by two TMS measures: resting motor threshold (RMT), an index of general neuronal membrane excitability, and motor-evoked potential (MEP) size, an index of global corticospinal pathway excitability (35–37). LTP-like plasticity is assessed by an increase in cortical excitability reflected by a reduction in RMT and facilitation of MEP size; the reverse is true for LTD-like plasticity (17).

A TMS protocol used for the induction of long-lasting cortical plasticity in the motor cortex is repetitive transcranial magnetic stimulation (rTMS). The direction of cortical excitability change following rTMS is dependent on the frequency and pattern of stimulation (38, 39). Studies in healthy subjects show that low frequency rTMS (≤1 Hz) induces LTD-like plasticity, whereas high frequency rTMS (≥5 Hz) induces LTP-like plasticity (11, 39). Large inter-individual variability of the physiological response to high and low-frequency rTMS has been observed. This can be attributed to several factors that impact cortical plasticity such as age, gender, attention level, and genetics (38).

To date, two studies have used rTMS to investigate plasticity deficits in SCZ. The first study investigated LTD-like plasticity following 1-Hz rTMS over the left primary motor cortex in 10 medicated and 16 unmedicated SCZ patients compared to 18 healthy controls (36). Both patient groups failed to demonstrate LTD-like plasticity, assessed by a failure to increase the RMT, as compared to the control group. Both patient groups also exhibited reduced cortical inhibition, demonstrated by a decrease in GABAergic neurotransmission which is indexed by paired-pulse and single-pulse TMS protocols. An association between the reduced plasticity response and cortical inhibition was also found (36). Together these results show impaired LTD-like plasticity and deficient cortical inhibition regardless of medication status in SCZ patients, indicative of a disease-dependent impairment of LTD-like plasticity in SCZ. There is evidence expanding the findings of altered cortical inhibition in SCZ to areas outside of the motor cortex. Woo et al. (40) and Kalus et al. (41) show altered cortical inhibition within the anterior cingulate cortex (ACC), a brain region implicated in the pathophysiology of SCZ. Investigators also suggest cortical inhibition is likely to play a crucial role in the physiologic basis of the observed plasticity deficits.

A second study used the same 1-Hz rTMS protocol to investigate intracortical connectivity in SCZ by stimulating the left premotor cortex to assess ipsilateral cortical excitability changes in the left primary motor cortex (42). Healthy controls displayed LTD-like plasticity indexed by a reduction in cortical excitability following 1-Hz rTMS. This result is consistent with previous studies in healthy subjects that also demonstrated a suppression of motor cortical excitability following 1-Hz rTMS over the premotor cortex (43, 44). Conversely, SCZ patients demonstrated LTP-like plasticity as opposed to LTD. SCZ patients also showed cortical inhibition deficits compared to controls (42). This study provides indirect evidence for a close association between dysfunctional plasticity, reduced cortical inhibition, and functional premotor–motor connectivity in SCZ patients.

### Paired Associative Stimulation

Paired associative stimulation (PAS) is another TMS paradigm used to induce reversible LTP and LTD-like cortical plasticity. It requires pairing of slow-rate, repetitive, low-frequency electrical stimulation of the peripheral median nerve with TMS stimulation of the contralateral motor cortex. The electrical stimulus precedes the TMS stimulus by either 25 ms to induce LTP or by 10 ms to induce LTD (45, 46). One study investigated PAS-induced LTP-like plasticity and its association with motor skill learning in 15 medicated patients suffering from SCZ or schizoaffective disorders and 15 healthy controls (47). In contrast to controls SCZ patients failed to demonstrate LTP-like plasticity assessed by a failure to increase MEP size. Motor skill learning was evaluated using a rotary pursuit task. The task required subjects to track a spot target on a revolving wheel with a stylus. On repeated exposure, the total amount of contact time on target per trial was used as an index of motor learning (47). The investigators found LTP-like plasticity and motor skill learning to be positively correlated among patients and controls. This finding highlights the close association between impaired plasticity and learning and memory deficits in SCZ patients.

### Use-Dependent Plasticity

Use-dependent plasticity (UDP) is a TMS protocol that enables evaluation of motor cortical reorganizational processes thought to index a form of neuroplasticity (9). The neurobiological basis of cortical excitability changes induced by UDP are similar to that of LTP. Pharmacological studies in healthy subjects have shown a reduction in UDP after administration of dextromethorphan (NMDA receptor blocker) and lorazepam (GABA_A_ receptor positive allosteric modulator) (25, 48). The UDP protocol involves measuring the spontaneous direction of TMS-induced thumb movement before and after a 30-min training period in which participants practice thumb movements in the opposite direction of baseline. Healthy subjects have demonstrated TMS-induced thumb movements in the direction of training immediately after training; this effect has been shown to taper off within 40 min (49).

Only one study has investigated UDP in SCZ patients. This study included 14 medicated, 6 unmedicated SCZ patients, and 20 healthy controls (9). In this study both patient groups failed to reorient TMS-induced thumb movements in the direction of training, the controls did not. The failure to reorient thumb movements could not be accounted for by training differences between groups (9). These results provide additional neurophysiological evidence for plasticity deficits in SCZ, independent of their medication status, emphasizing the link between disturbed plasticity and motor learning deficits in SCZ patients.

### Transcranial Direct Current Stimulation

Cortical excitability modulation using the aforementioned TMS protocols is dependent on depolarization of cortical neurons in response to a suprathreshold TMS pulse. Transcranial direct current stimulation (tDCS) involves applications of continuous low direct current via two electrodes (anodal and cathodal) to induce intracortical current flow and subsequent modulation of the neuronal resting membrane potential (7, 27). The direction of excitability changes is dependent upon the stimulation protocol used; the duration of excitability changes is dependent on the length and intensity of stimulation (27, 50, 51). For anodal tDCS the anodal electrode is placed over the motor cortex and the cathodal electrode is placed over the contralateral orbit as a reference; this placement is reversed for cathodal tDCS (27). In the summarized studies below 9 min of cathodal tDCS at an intensity of 1 mA was used to induce LTD-like plasticity, and 13 min of anodal tDCS at an intensity of 1 mA was used to induce LTP-like plasticity in the motor cortex of SCZ patients.

A cross-sectional study explored non-focal LTP-like plasticity following anodal tDCS in the left primary motor cortex of 9 recent-onset SCZ, 13 multi-episode SCZ patients, and 22 healthy controls (52). Only the multi-episode group displayed deficient LTP-like plasticity when compared to recent-onset SCZ patients and healthy controls. Both patient groups also exhibited cortical inhibition deficits but only the recent-onset group demonstrated an enhancement of cortical inhibition. These findings underscore a possible link between duration and severity of SCZ and impaired plasticity (52). This has significant implications for the use of NIBS techniques as both an investigatory and therapeutic tool.

LTD-like plasticity following cathodal tDCS has been extensively explored in the motor cortex of SCZ patients. The first of these studies examined the LTD-like plasticity aftereffects of cathodal tDCS in the left primary motor cortex of 21 SCZ patients and 21 healthy subjects (53). In this study SCZ patients demonstrated abolished LTD-like plasticity and alterations in cortical inhibition compared to controls. This finding of deficient LTD-like plasticity in the left primary motor cortex of SCZ patients was successfully replicated in three subsequent studies by the same group. In addition to replicating this finding, these studies provided indirect evidence for the relationship between impaired plasticity and dysfunctional interhemispheric connectivity in SCZ patients. For instance, SCZ patients demonstrated reduced LTD-like plasticity in the stimulated (left) and abolished LTD-like plasticity in the non-stimulated (right) hemisphere following cathodal tDCS to the left primary motor cortex (54). Additionally a pilot study, using the same cathodal-tDCS protocol, provided preliminary neurophysiological evidence for the impact of a genetic liability for SCZ on LTD-like plasticity (55). In this study unaffected and unmedicated first-degree relatives of SCZ patients also demonstrated abolished LTD-like plasticity in the stimulated hemisphere and an unexpected reversal of plasticity in the non-stimulated hemisphere. This finding further supports the trait-dependent impairment of neuroplasticity in SCZ. There are several possible physiological mechanisms that could account for the observed reversal of cortical excitability. The investigators speculate that the observed plasticity could be due to deficient interhemispheric inhibition, an imbalance in inhibitory interhemispheric M1-to-M1 connection within the plasticity–connectivity network, or homeostatic mechanisms (55). More recently, one proof-of-concept study investigated the efficacy of bilateral tDCS (cathode left M1, anode right M1) in SCZ patients (56). The premise of this study was based on the recent finding of LTP- and LTD-like alterations in cortical excitability on the anodal and cathodal stimulation sites following bilateral tDCS in healthy subjects (57). This result was not replicated in this study, as healthy controls only demonstrated LTP-like plasticity induction following bilateral stimulation. In contrast to controls, SCZ patients failed to demonstrate both LTP- and LTD-like plasticity following bilateral tDCS. The consistent LTD-like plasticity deficits observed in the aforementioned studies are suggested to play a crucial role in the pathophysiology of cognitive and memory deficits in SCZ (7).

### Theta-Burst Stimulation

Theta-burst stimulation (TBS), a recently developed rTMS protocol, has also been used to induce cortical plasticity. Using high frequency stimulation, TBS modulates cortical excitability; the direction of cortical excitability change is dependent on the pattern of stimulation used. TBS requires a significantly reduced stimulation period compared to the standard rTMS protocols discussed previously (58, 59). Intermittent TBS (iTBS) involves the delivery of short 2 s trains repeated at 10 s intervals for 20 cycles; continuous TBS (cTBS) involves a single stimulation train of 40 s. These stimulation protocols induce LTP- and LTD-like plasticity respectively (58, 59). The LTP- and LTD-like plasticity changes following TBS are shown to be critically dependent on: NMDA receptors, calcium homeostasis, and the balance between excitatory and inhibitory interneuronal networks (59–62).

A proof-of-concept study investigated TBS-induced LTP and LTD-like plasticity in the left primary motor cortex of 10 SCZ patients and 10 healthy controls (60). In healthy subjects cTBS300 and cTBS600 induce LTP- and LTD-like cortical excitability changes in the motor cortex (58–60, 63). The duration of the stimulation protocol determines the direction of cortical plasticity changes; cTBS300 delivers high frequency stimulation in a continuous train lasting 20 s, whereas cTBS600 lasts 40 s (60). As expected, SCZ patients did not show LTD-like plasticity unlike healthy controls. Following cTBS300 SCZ patients demonstrated reverse plasticity in contrast to healthy controls who demonstrated a numeric, but non-­significant, increase in cortical excitability. In total a larger extent of healthy subjects than SCZ patients displayed the expected plastic response following both cTBS paradigms (60). These preliminary findings provide additional evidence for dysfunctional cortical plasticity in SCZ patients.

## Discussion

The reviewed articles generated three main themes. First, all studies demonstrated disturbed cortical plasticity in SCZ patients regardless of the investigatory NIBS technique used. Second, glutamatergic NMDA receptor dysfunction and alterations in GABAergic neurotransmission are speculated to play a key role in the neurobiological mechanisms underpinning the observed plasticity deficits in SCZ. It is well known that enhanced dopaminergic transmission is implicated in the pathophysiology of SCZ. Altered dopamine transmission has been shown to modulate glutamate and GABA among other neurotransmitter systems (18). The dopamine-dependent modulation of cortical plasticity is complex and further complicated in SCZ patients due to antipsychotic use (53). Third, the summarized studies lend support to the dysconnectivity hypothesis by providing indirect evidence for plasticity-dependent inter- and intra-hemispheric cortical connectivity disturbances. This hypothesis, set forth by Stephan et al. (5), postulates that dysfunction of NMDA receptors, and subsequent impairment of NMDA receptor-mediated plasticity, impacts long-range connections in the developing brain. As a result of these developmental aberrations, learning and memory processes can become impaired as they require precise control of synaptic activity and inhibition (17).

LTD plays a crucial role in regulating the signal-to-noise ratio and memory functions in the human brain (64, 65). The reduced cortical inhibition and deficient LTD-like plasticity demonstrated by SCZ patients in the reviewed studies lends support to the theory of a reduced signal-to-noise ratio and disturbed filter-function in SCZ (7, 52). A reduction in cortical inhibition may cause an enhancement of cortical noise, resulting in decreased spike-timing-dependent plasticity and non-focal plasticity. As a corollary, enhancement of cortical nose may cause a reduction in the signal-to-noise ratio and filter-function of the brain. This is thought to underlie the observed cognitive deficits and memory impairments in SCZ patients (7, 52).

Apart from being used as an investigatory tool to study the neurophysiological and neurobiological underpinnings of SCZ, NIBS has also been used as a therapeutic tool. However, clinical trials using NIBS techniques to treat SCZ symptoms, such as treatment-refractory auditory hallucinations, cognitive deficits, and negative symptoms have produced both promising and equivocal results (66–70). The following three major unsolved issues must be addressed before NIBS becomes a routine clinical application in the treatment of SCZ. First, optimal stimulation protocols need to be established (11). In this regard, rTMS- and tDCS-based protocols may be preferred to PAS and UDP because these protocols are easier to use in a clinical setting. Future studies comparing different stimulation techniques are needed to address this issue (11). Second, the optimal cortical region must be determined for the target symptom. Several brain regions are implicated in the pathophysiology of SCZ such as the prefrontal cortex, temporoparietal cortex, and the cerebellum. It has been discussed that the symptomatology of the patient should determine the area of stimulation (11). For instance, targeting the temporoparietal cortex for treatment-resistant auditory hallucinations (71). Finally, consideration must be given to the duration of illness when considering NIBS. Evidence exists for a process of progressive brain change, indexed by tissue volume decrease after onset in SCZ patients (72). Additional neurophysiological evidence suggests a link between chronicity of SCZ and disturbed plasticity (52). For future treatment intervention during early phases (i.e., first-episode patients) appears to be a promising target.

Several limitations of this review warrant further discussion. First, apart from the cathodal-tDCS studies, the majority of studies included here have not been replicated in large and independent samples (11). A second limiting factor is the impact of antipsychotic medication on cortical plasticity induction following NIBS. The mechanism of action for atypical antipsychotics and neuroleptics is critically dependent on the modulation of dopaminergic transmission. Pharmacological studies using single administration of dopamine agonists and antagonists in healthy subjects demonstrate the crucial role of dopamine levels on cortical plasticity induction following PAS, tDCS, and UDP protocols (11, 48, 53). D2-receptor antagonism has been shown to eliminate both anodal-tDCS and cathodal-tDCS plasticity aftereffects (73). Additionally, several tDCS studies have shown a non-linear and dose-dependent effect of dopamine modulation on cortical excitability and plasticity (11, 52).

The dopamine receptor antagonist haloperidol has been demonstrated to suppress plasticity induction following both PAS and UDP protocols (48). Apart from dopamine, pharmacological alterations in other neuromodulators such as catecholamines, acetylcholine, and serotonin have also been observed to modulate cortical plasticity induction following tDCS, PAS, and UDP protocols (11, 48). The effects of pharmacological modulation on PAS- and tDCS-induced plasticity have been explored to a greater extent than rTMS (11). Even though the mechanisms that underlie plasticity aftereffects of PAS, tDCS and rTMS may not be identical, it is very likely that modulation of the aforementioned neuromodulatory systems will also impact rTMS-induced plasticity (11, 48).

One should note that the cited studies discussed in the preceding paragraph investigated drug administration in healthy subjects. The translation of these studies’ findings to SCZ patients is debatable. First, SCZ patients and healthy subjects have a different distribution of neurotransmitters and their related receptors. It is well known that enhanced dopaminergic transmission is implicated in the pathophysiology of SCZ (74). Hence antipsychotics are more likely to normalize dopamine levels in SCZ patients rather than induce a hypodopaminergic state as they would in healthy subjects (53, 56). Second, studies have largely focused on acute drug effects, usually single-dose administration. It is very likely that these studies may not reflect the pharmacokinetic and pharmacodynamic properties of chronic antipsychotic use in SCZ patients (53) and definitely do not reflect clinical practice. Studies have also demonstrated a link between chronic exposure to antipsychotic medication and volumetric brain changes (75–77), suggesting that single-dose medication studies do not capture the long-term changes that accompany chronic SCZ. Further research is needed to address the impact of acute versus chronic antipsychotic use on NIBS-induced plasticity.

Two NIBS studies have demonstrated disturbed cortical plasticity in both medicated and unmedicated SCZ patients (9, 36). In another study, unaffected and unmedicated first-degree relatives of SCZ patients also showed abolished LTD-like plasticity following cathodal-tDCS (55). These findings underscore the fact that antipsychotic medications may account for a part of the cortical plasticity disturbances observed in SCZ patients but they cannot account for it all.

An intrinsic limitation in the methodology of the reviewed studies is the difficulty of translating the results from the motor system (premotor and primary motor cortex) to other parts of the brain that may be more associated with the pathophysiology of SCZ. For example, the dorsolateral prefrontal cortex is an area involved in cognitive and executive functioning known to be impaired in SCZ. The decision to stimulate the motor cortex to explore cortical plasticity in SCZ patients was based on the use of TMS and surface electromyogram to measure cortical excitability before and after the administration of NIBS protocols. Variations in cortical architecture, neurotransmitter distribution, and receptor density exist between brain regions outside of and those comprising the motor system (11). It is possible that these fundamental neurobiological differences between brain regions may result in a different pathophysiological response to NIBS compared to that elicited from the motor cortex (11). However, it is important to note that neuropathological studies in SCZ patient have shown that alterations in the primary motor cortex are related to the dorsolateral prefrontal cortex and ACC (11, 20). In addition, a separate study has provided neurophysiological evidence for a correlation between the extent of cortical inhibition in the motor cortex and the dorsolateral prefrontal cortex in healthy subjects (78). These studies provide evidence supporting the translatability of motor cortical studies to other brain regions. However, further work still needs to be done in other brain regions using neuroimaging techniques due to factors such as cortical thickness, neuron density, and cortical excitability that may differ between regions.

Recent technological advancements have allowed investigators to combine neuroimaging techniques, such as the electroencephalogram (EEG) and functional magnetic resonance imaging, with NIBS methods to investigate cortical excitability, plasticity, and connectivity outside of the motor system, bypassing this intrinsic limitation of NIBS techniques (11). For instance, using a PAS–EEG protocol, Rajji et al. (79) were able to effectively induce plasticity in the dorsolateral prefrontal cortex and measure the output using cortical-evoked activity instead of MEP. Future studies using a combination of neuroimaging and NIBS techniques have the potential to index plasticity from regions more closely associated with the pathophysiology of SCZ, allowing us to study SCZ in a more clinically relevant manner.

Another limitation is the influence of disease course and severity on NIBS-induced cortical plasticity. Longitudinal neuroimaging studies have emphasized a neuroprogressive component of SCZ. In one such study, a subset of SCZ patients showed a progressive postonset reduction in several gray and white matter regions, with greatest severity in the frontal lobes and a concomitant increase in cerebrospinal fluid in the lateral ventricles and frontal, temporal, and parietal sulci (72). Another study showed an association between longer durations of psychosis, gray matter volume loss, ventricle volume increases, and greater reduction in total brain and cerebellar volume (80). This has important implications for using NIBS to investigate cortical plasticity in SCZ patients as there is a close association between cortical thickness and the extent of NIBS-induced plasticity (81). One study has shown a positive correlation between cortical thickness and the extent of PAS-induced LTP-like cortical excitability changes in healthy subjects. Subjects with thicker gray matter in the left sensorimotor cortex exhibited stronger PAS aftereffects than those with thinner gray matter (81). These findings emphasize the importance of addressing the duration and severity of SCZ when using NIBS protocols.

Several other determinants influencing NIBS-induced cortical plasticity have been identified. These include gender, physical activity, and attention (38, 81) – an extensive analysis of the limitations is beyond this paper’s scope. These, among several other important issues, must be considered in the design and execution of future NIBS studies that seek to elucidate the pathophysiology of SCZ.

## Conclusion

This review summarizes the neurophysiological evidence for disrupted motor cortical plasticity in SCZ, regardless of medication status, and its close association with alterations in cortical inhibition and dysfunctional intracortical connectivity. Dysfunction of glutamatergic NMDA receptors and alterations in GABAergic neuronal networks comprise part of the larger neurobiological framework underpinning the observed plasticity deficits in SCZ. NIBS provide a valuable avenue toward elucidating the neurobiological mechanisms that underlie dysfunctional plasticity in SCZ.

## Author Contributions

AB and DB were responsible for the completion and design of the review. AB acquired the data and AB and DB analyzed and interpreted the data and drafted the manuscript. All authors revised the manuscript critically for important intellectual content. All authors approved the version of the manuscript to be published.

## Conflict of Interest Statement

AB does not have any financial disclosures. DV is partially supported by a Lilly American Psychiatric Research Fellowship. TR receives research support from Brain Canada and Behavior Research Foundation, Canadian Foundation for Innovation, Canadian Institutes of Health Research (CIHR), Ontario Ministry of Health and Long-Term Care, Ontario Ministry of Research and Innovation, the US National Institute of Health (NIH), and the W. Garfield Weston Foundation. TR reports no competing interests. ZD received external funding through Brainsway Ltd. and a travel allowance through Pfizer and Merck. ZD has also received speaker funding through Sepracor Inc., AstraZeneca and served on the advisory board for Hoffman-La Roche Limited. ZD has received funding from the Ontario Mental Health Foundation (OMHF), CIHR, the Brain and Behaviour Research Foundation, and the Temerty Family and Grant Family and through the CAMH Foundation and the Campbell Institute. DB receives research support from the Canadian Institutes of Health Research (CIHR), Brain Canada, National Institutes of Health (NIH), Temerty Family through the Centre for Addiction and Mental Health (CAMH) Foundation and the Campbell Family Research Institute. He receives non-salary operating funds and in-kind equipment support from Brainsway Ltd., for an investigator-initiated study. He is the site principal investigator for several sponsor-initiated clinical trials from Brainsway Ltd. He receives in-kind equipment support from Tonika/Magventure for an investigator-initiated study.
